# Genetic Mapping by Sequencing More Precisely Detects Loci Responsible for Anaerobic Germination Tolerance in Rice

**DOI:** 10.3390/plants10040705

**Published:** 2021-04-06

**Authors:** John Carlos I. Ignacio, Maricris Zaidem, Carlos Casal, Shalabh Dixit, Tobias Kretzschmar, Jaime M. Samaniego, Merlyn S. Mendioro, Detlef Weigel, Endang M. Septiningsih

**Affiliations:** 1 International Rice Research Institute, DAPO Box 7777, Metro Manila 1301, Philippines; ignacio.8@osu.edu (J.C.I.I.); c.casal@irri.org, (C.C.J.); s.dixit@irri.org (S.D.); Tobias.Kretzschmar@scu.edu.au (T.K.); 2 University of the Philippines, Los Baños, Laguna 4031, Philippines; jmsamaniego2@up.edu.ph (J.M.S.); msmendioro@up.edu.ph (M.S.M.); 3 Department of Molecular Biology, Max Planck Institute for Developmental Biology, 72076 Tübingen, Germany; mzaidem@gmail.com (M.Z.); detlef.weigel@tuebingen.mpg.de (D.W.); 4 Department of Horticulture and Crop Science, Ohio Agriculture Research and Development Center, The Ohio State University, 1680 Madison Ave, Wooster, OH 44691, USA; 5 Department of Plant Sciences, University of Oxford, Oxford OX1 3RB, UK; 6 Southern Cross Plant Sciences, Southern Cross University, 1 Military Road, Lismore, NSW 2480, Australia; 7 Department of Soil and Crop Sciences, Texas A&M University, College Station, TX 77843, USA

**Keywords:** quantitative trait locus (QTL), sequencing based genotyping, RAD-seq, anaerobic germination (AG), rice (*Oryza sativa*)

## Abstract

Direct seeded rice (DSR) is a mainstay for planting rice in the Americas, and it is rapidly becoming more popular in Asia. It is essential to develop rice varieties that are suitable for this type of production system. ASD1, a landrace from India, possesses several traits desirable for direct-seeded fields, including tolerance to anaerobic germination (AG). To map the genetic basis of its tolerance, we examined a population of 200 F_2:3_ families derived from a cross between IR64 and ASD1 using the restriction site-associated DNA sequencing (RAD-seq) technology. This genotyping platform enabled the identification of 1921 single nucleotide polymorphism (SNP) markers to construct a high-resolution genetic linkage map with an average interval of 0.9 cM. Two significant quantitative trait loci (QTLs) were detected on chromosomes 7 and 9, *qAG7* and *qAG9*, with LOD scores of 7.1 and 15.0 and R^2^ values of 15.1 and 29.4, respectively. Here, we obtained more precise locations of the QTLs than traditional simple sequence repeat and low-density SNP genotyping methods and may help further dissect the genetic factors of these QTLs.

## 1. Introduction

Farmers have increasingly adopted direct seeded rice (DSR) in both rainfed and irrigated ecosystems since it requires less labor and cost of production compared to conventional transplanting [1]. However, unleveled fields or heavy rainfall on direct-seeded fields can lead to poor crop establishment as most rice varieties are susceptible to flooding during germination or anaerobic germination (AG). Hence, breeding for DSR requires tolerance to AG stress and is essential to sustain rice production in these ecosystems. Screening of over 8000 gene bank accessions and breeding lines at the International Rice Research Institute (IRRI) identified only 19 tolerant lines with greater than 70% survival rate [2]. Additional efforts to screen more varieties had been conducted at IRRI (E. Septiningsih, unpublished data). Some of these accessions have been used for physiological and agronomical studies, and as donors in genetic studies and crop improvement efforts [2,3,4,5,6,7,8,9,10,11,12,13,14,15,16,17].

Several biparental quantitative trait loci (QTL) mapping studies for AG tolerance have been performed previously using various genotyping platforms. For example, a study by Jiang et al. [18] reported five QTLs for AG using restriction fragment length polymorphism (RFLP) markers on a mapping population derived from Kinmaze/DV85. Using 135 simple-sequence repeat (SSR) markers, Angaji et al. [2] identified five QTLs, including *qAG-9-2* or *AG1*, on a BC_2_F_2_ population derived from IR64/Khao Hlan On. *AG1* has been used in breeding efforts (Toledo et al. 2015) and the gene underlying this QTL had been cloned as trehalose-6-phosphate phosphatase (*OsTPP7*) [14]. Six QTLs were identified in a study by Septiningsih et al. [11], including a major QTL on chromosome 7, derived from an F_2:3_ population of IR42/Ma-Zhan Red, using 118 SSR markers. Baltazar et al. [12] identified three QTLs, including the major one on chromosome 7, derived from an F_2:3_ population of IR64/Nanhi, using 384-plex single nucleotide polymorphism (SNP) Indica/Indica set on the Illumina BeadXpress Reader, resulting of 234 SNP markers used in the map. Additionally, using the same genotyping platform but with an F_2:3_ population derived from IR64/Kharsu 80A, resulting in 217 polymorphic markers, Baltazar et al. [13] identified four QTLs, of which three of them were detected on chromosome 7. More recently, Ghosal et al. [4] identified five QTLs on a BC_1_F_2_:_3_ mapping population of NSIC Rc238/Kalarata using 185 kompetitive allele specific PCR (KASP) SNP markers. Using the same genotyping platform of 189 KASP SNP markers and the same donor, Kalarata, but a different elite cultivar, NSIC Rc222, the team identified four QTLs using a BC_1_F_2_:_3_ mapping population. Three common QTLs on chromosomes 3, 6, and 7, were detected across the two populations. Additionally, Ghosal et al. [9] also reported four AG QTLs from a BC_1_F_2_:_3_ mapping population derived from NSIC Rc222/BJ1 through bulk-segregant analysis strategy using 102 SSR markers. While these studies have pointed to a number of significant QTLs for AG tolerance in rice, their limited marker resolution has made it challenging to narrow down the QTL regions. 

Exploration of simple, inexpensive high-density scalable single nucleotide polymorphism (SNP) genotyping led to the development of sequencing of restriction site-associated DNA (RAD) tags [19]. This RAD sequencing (RAD-seq) approach utilizes one or two restriction enzymes to digest genomic DNA samples at many short identical sites so that the resulting fragments can be uniquely barcoded. Barcoding allows samples to be multiplexed in a library and genotyped together in a single sequencing run. Elshire et al. [20] have also reported a similar approach called Genotyping-by-Sequencing (GBS). Sequencing-based genotyping is so robust that it can be used in a wide range of genetic studies, such as genome-wide association studies (GWAS), genomic selection (GS), diversity studies, and genome mapping, even without prior knowledge of the species [19,21]. QTL mapping using RAD-seq as a genotyping platform has been performed in various crops, including soybean [22,23,24,25], peanut [26,27,28], cowpea [29], bitter gourd [30,31], Chinese cabbage [32], alfalfa [33], sesame [34], foxtail millet [33,35], sorghum [36], and rice [37,38].

ASD1, an *Indica* rice variety from India [39], has been characterized for several traits desired for DSR, including AG tolerance, and found to be a promising genetic donor for this trait [40]. In this study, we aim to map the genetic factors responsible for AG tolerance from ASD1 with the use of a biparental F_2_ population and RAD-seq technology as the genotyping platform. 

## 2. Results

### 2.1. Phenotypic Performance

**Figure 1:**
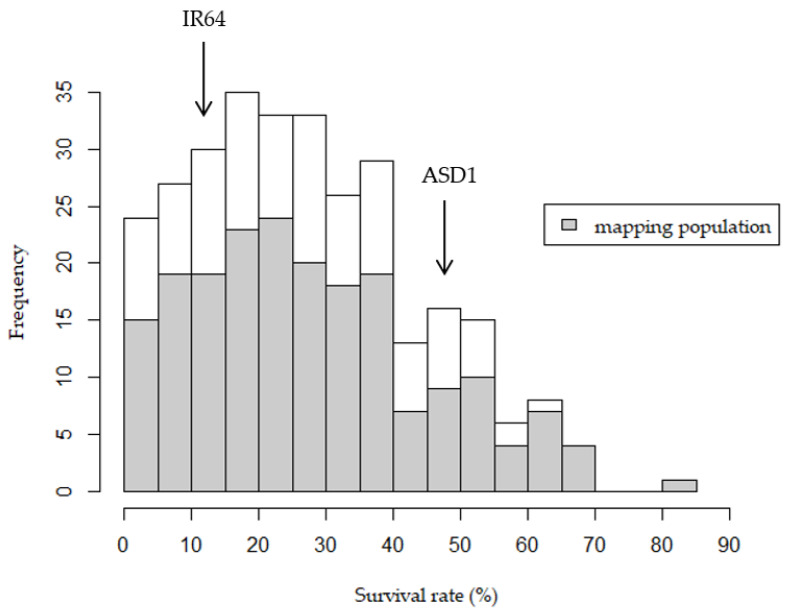
Survival rates of the 300 IR64 × ASD1 F_2:3_ families under anaerobic germination, along with the average survival of the parents. The 199 families used in quantitative trait loci (QTL) mapping are shown in grey.

From the 418 randomly selected families and parental lines tested for germination rate under normal conditions, 118 had germination rates of 60 to 97%, while 300 families had germination rates higher than 97%, which were then tested for survivability under AG. The parental controls also showed 97–100% germination rates under normal conditions. Among the F_2:3_ families, the survival rates under AG were ranging between 0% and 83%. The average survival rates of the parents, IR64 and ASD1, under AG were 11.67% and 46.55%, respectively (Figure 1; Figure S1). The mapping population demonstrated transgressive segregation in both directions, where 60 out of 300 families (20%) had lower survival rates than that of IR64, while 50 out of the 300 families (17%) had higher survival rates than that of ASD1. IR64 is considered moderately sensitive to AG compared to other sensitive accessions [2], and, even in the case of mapping populations made with a highly sensitive accession, such as IR42, transgressive segregation could still be observed [11].

### 2.2. Genotyping

#### 2.2.1. Library Preparation and Sequencing

All extracted DNA samples had 260/230 absorbance ranging from 1.75 to 1.85 and had 260/230 absorbance of 1.8 to 2.2. Based on the Agilent 2100 Bioanalyzer DNA 1000 chip results, each of the libraries had concentrations of around 300 pg/mL and fragment size ranging 300–600 bp. Each sequencing lane (also each library) produced roughly 380 million reads with length of 101 bp and an average GC content of 45%.

#### 2.2.2. Variant Detection

For each of the three raw sequencing outputs, about 200 million duplicated reads were removed. About 80% of the total reads were demultiplexed, and the average read count was 1.54 million per sample, with only 32 samples having less than 100,000 reads. The combination of the 3 replicated parents from all sequencing lanes resulted in 3.02 million reads assigned to IR64, while 4.19 million reads to ASD1. On average, about 97% of the deduplicated and demultiplexed reads from each sequencing lane aligned with the reference genome. A total of 156,636 variants were discovered in 270 F_2:3_ families and two parental lines. Among these, 137,819 were SNPs, and 18,817 were indels.

#### 2.2.3. Variant and Sample Filtering

All 18,817 indels and 1659 multiallelic SNPs (allele count > 2) were filtered out of the dataset. Out of the 136,160 remaining SNPs, only 6068 were homozygous and identified to be polymorphic between the two parents. SNPs with less than 0.18 minimum allele frequencies and heterozygous proportions less than 0.05 and greater than 0.55 were excluded from the dataset, removing 321 sites. After removing SNPs from the same reads, the number of SNPs was further reduced by 2007. By taking only samples and markers with call rates of at least 50% and 60%, respectively, the dataset had a remaining total of 200 samples and 3436 markers and was used for imputation and genotyping call correction.

#### 2.2.4. Adapting Genotyping Data for F_2_ Genetic Analysis

Missing data proportion was greatly reduced from 20.4% to 6.1% after imputation (Table S1). A closer to 1AA:2AB:1BB Mendelian genotype F_2_ ratio was attained after correcting for under-called heterozygous genotypes. The remaining steps did not greatly affect the distributions of genotypes but influenced the estimation of the genetic map where initial estimated maps without correction reached more than 1000 cM per chromosome (data not shown). Furuta et al. [41] noted that, when GBS data on a rice F_2_ population was left uncorrected, the genetic map of a chromosome can reach up to 3500 cM. In their case, correction reduced total map size of all chromosomes to 1536 cM, which was similar to our observation.

### 2.3. QTL Analysis

#### 2.3.1. Genetic Map Estimation

**Figure 2:**
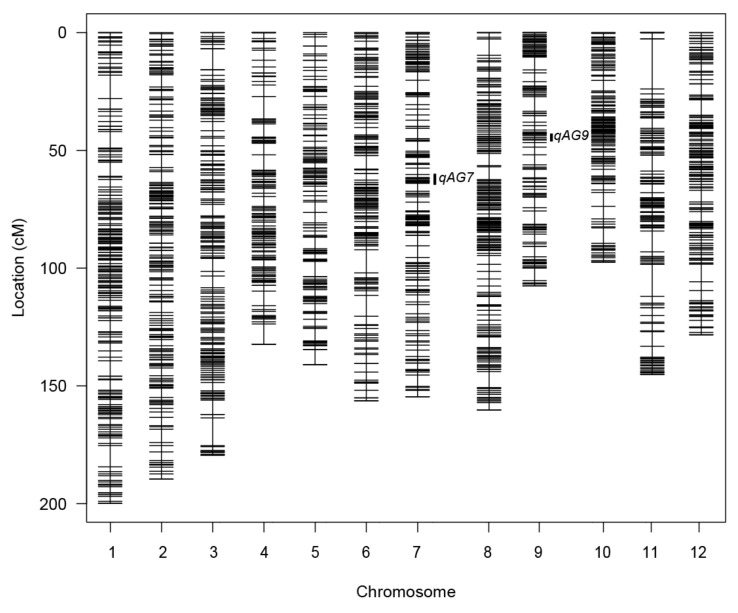
Estimated genetic map of IR64/ASD1 population constructed from 200 F_2:3_ families using 1921 single nucleotide polymorphism (SNP) markers. Each chromosome is represented by a vertical line and each marker by a horizontal line on its respective chromosome position. The positions of the QTLs detected on chromosomes 7 and 9 are represented by black vertical bars.

Before map estimation, 1515 SNP markers contained duplicate information and were removed from the dataset. The map was estimated using 1921 markers and 200 F_2:3_ families, resulting in a total length of 1791.5 cM with an average of 0.9 cM distance between markers. The biggest gap of 21.2 cM was found on chromosome 11 (Table 1 and Figure 2), which may be attributed to lack of polymorphic SNPs or over filtering of variants within this region.

#### 2.3.2. QTL Detection

Out of the 300 F_2:3_ families that were phenotyped, only 199 had genotype data. The two datasets were intersected, resulting in a combined dataset of 199 families and 1921 markers used for QTL detection. Logarithm of the odds (LOD) thresholds for 0.05 and 0.01 significance levels were established at 3.92 and 4.76 for interval mapping (IM) and 3.95 and 4.83 for composite interval mapping (CIM) using 10,000 permutations in QGene [42]. Similarly, the LOD thresholds were 3.85 and 4.69 for IM and 3.86 and 4.74 for CIM using QTL Cartographer with 1000 permutations [43]. Based on the analyses, two significant QTLs with LOD scores above the permutation thresholds (*p* ≤ 0.01) were identified, and in all cases, the tolerant alleles came from ASD1. These two QTLs, located on the long arm of chromosome 9, *qAG9*, and the short arm of chromosome 7, *qAG7*, were consistently detected by IM and CIM using QGene and QTL Cartographer (Figure 2, Table 2, Figures S2 and S3). The detection by QGene CIM had an LOD score of 15.0 and explains phenotypic variance (R^2^) of 29.4 % for *qAG9*, while it reported an LOD score of 7.1 and R^2^ of 15.1 % for *qAG7*. Two minor QTLs, with LOD scores slightly below the permutation threshold, were detected on chromosome 5, *qAG5,* with LOD score of 2.8 and R^2^ of 5.4%; and one on chromosome 6, *qAG6*, with LOD score of 2.3 and an R^2^ of 5.1%. These two minor QTLs were also derived from ASD1 (Table 2). 

## 3. Discussion

Four AG QTLs were identified in the QTL analysis, with two above the permutation threshold on chromosomes 7 (*qAG7*) and chromosome 9 (*qAG9*). The QTL with the most statistical support and largest effect, *qAG9*, was detected in the 12.03–12.33 Mb region of chromosome 9 with a peak at 12.28 Mb and is co-located within the *qAG-9-2* region, 11.75–12.55 Mb, as reported by Angaji et al. [2] (Table 3). Kretzschmar et al. [14] have reported, and confirmed by map-based cloning, that a *trehalose-6-phosphate phosphatase* gene, *OsTPP7*, is the causal gene underlying *qAG-9-2*. *OsTPP7* has a locus identifier LOC_Os09g20390 and is reported to be positioned exactly at 12.25 Mb of chromosome 9 according to the rice genome annotation project [44]. Both *qAG9* and *qAG-9-2* were the QTLs with the most statistical support and largest effect detected in their corresponding studies, both having LOD scores greater than the *p* ≤ 0.01 significance thresholds and explaining phenotypic variance of 20–33%. In addition, both studies used IR64 as the sensitive parent in the development of the mapping population. However, unlike in the report of Angaji et al. [2], no QTL was detected from IR64 in this current study. 

The second QTL with the most statistical support and largest effect detected in this study, *qAG7*, was positioned between 6.30–7.65 Mb of chromosome 7, with a peak LOD score at 7.55 Mb. Several other studies have also reported AG tolerance QTL on chromosome 7. A report from Ghosal et al. [4] identified *qSUR7–1_Rc238-SCR-21_* from the accession Kalarata with a peak at 6.07 Mb (Table 4). There is no information about the flanking region of *qSUR7–1_Rc238-SCR-21_*, but it seems that the QTL region spans a large interval on chromosome 7 based on the reported graphs. Similarly, the studies of AG tolerance in the reported *qAG7* from Nanhi [12] located at 4.80–18.48 Mb, and in the reported *qAG-7*-1 from Khao Hlan On [2] located at 6.07–17.67 Mb span a wide range of QTL region of more than 10 Mb in chromosome 7. Three other QTLs for AG, *qAG7.1* from Ma-Zhan Red [11] located at 8.04 to 12.97 Mb, *qAG7.1* from Kharsu 80A [13] located from 3.68 to 6.01 Mb, and *qAGS-7* from TKM 9 [45] located from 7.61 to 12.78 Mb were reported to be in the similar region but do not overlap entirely with *qAG7*. Among all the studies that reported QTLs for AG in the short arm towards the centromeric region of chromosome 7, this study has shown the narrowest interval of the QTL spans (Table 4).

The similarities between the results of this study and that of Angaji et al. [2] for *qAG9* and *qAG-9-2* suggests that the same gene, *OsTPP7*, most likely controls their contributions to tolerance to AG, although this needs further confirmation. In contrast, although there have been many reported QTLs in the similar region of *qAG7*, there is not enough evidence currently to say whether *qAG7* and those previously identified QTLs located in the similar region of *qAG7* have the same genetic factor(s) responsible for AG tolerance, due to several non-overlapping regions. Thus, further investigation is needed.

In conclusion, it has been demonstrated in this study that the use of RAD-seq enables the construction of a high-resolution genetic linkage map with an average interval of 0.9 cM. This genotyping method may be used to obtain a precise approximation of QTLs compared to traditional SSR and low-density SNP genotyping methods, which can help us better understand the genetic basis of AG tolerance in rice. In this study, two significant QTLs for tolerance to anaerobic germination, *qAG7* and *qAG9*, were identified from ASD1. Compared to previous studies, these two QTLs showed more precise locations with the narrowest QTL regions. Though many factors can affect genetic mapping resolution, a higher number of markers can only improve the resolution to a certain extent. Increasing the population size, further backcrossing, or the use of recombinant inbred lines (RILs) can be done to achieve finer mapping resolution. However, these strategies usually require more time and resources. Whether the overlapping QTLs detected in this study and QTLs from other biparental mapping populations in the same chromosomal regions have the same causal gene(s) and/or alleles needs further investigation.

## 4. Materials and Methods

### 4.1. Development of Mapping Population

IR64 (GID 4483020), an *indica* mega-variety developed at the International Rice Research Institute (IRRI), which is susceptible to AG [2], was crossed with ASD1 (IRGC 6267; GID 328677), a landrace from India which is tolerant to AG [40,46]. The cross was made in IRRI, Los Baños, Laguna, Philippines in the 2012 wet season. In the 2013 dry season, F_1_ individuals were confirmed for true hybrids using SSR markers and were allowed to self-pollinate. In the 2015 dry season, 864 F_2_ progenies from a single F_1_ individual were harvested to produce F_2:3_ families for phenotyping. The seeds were oven-dried at 40 °C until moisture content was below 14%. The seeds were then stored at 10 °C for 5 months, awaiting phenotyping. Before phenotyping, seeds were incubated in 50 °C for 5 days to break dormancy, and 418 F_2:3_ randomly selected families were tested for germination rate together with parental lines using 40 seeds per entry at 30 °C incubator for 3 days. 

### 4.2. Phenotyping

Measurement of survivability under AG was conducted according to the protocol of Septiningsih et al. [11]. Each tray has 17x34 cells and can accommodate 11 entries with 30 seeds per entry sown at 1 cm depth. An entire column of cells between each entry and the outermost cells were left unseeded to minimize phenotyping error. The *DiGGer* R package [47,48] was used to generate 2 replications of alpha plus randomization design with the restriction of having 9 samples and 2 parental controls, ASD1 and IR64, in each tray. Each replicate was laid on an elevated concrete tank filled with tap water up to 10 cm above the tray’s surface. The water level was maintained up to 14 days, and then the numbers of seedlings that emerged out of the water were counted for each entry. The average survival of each entry was calculated from the two replicates and was used for the QTL study.

### 4.3. Genotyping

#### 4.3.1. Library Preparation and Sequencing

Genomic DNA from F_2_ leaf samples was isolated using cetyltrimethylammonium bromide (CTAB) extraction method at IRRI, Philippines [49]. DNA was then sent to the Max Plank Institute in Tübingen, Germany, where the DNA libraries were prepared and sequenced. DNA quality was checked with NanoDrop™ 8000 Spectrophotometer (Thermo Fisher Scientific, Waltham, MA, USA) using 260/230 and 260/280 ratios and quantified using Quant-It™ PicoGreen^®^ dsDNA (Thermo Fisher Scientific, Waltham, MA, USA) quantitation assay. After normalization of DNA concentration, three 96-plex DNA libraries were prepared with *KpnI* restriction enzyme. DNA barcodes for each library were selected from the 386-pool of *KpnI* optimized barcodes to obtain a GC content close to 50% to reduce sequencing errors. Barcoded DNA fragments for each library were multiplexed in a microcentrifuge tube and sheared with Covaris S220 Focused-ultrasonicator (Covaris, Woburn, MA, USA). End-repair and dA-tailing were performed after DNA shearing; then, universal sequencing adapters were ligated. After a PCR enrichment step, libraries were selected for 300–600 bp fragment size using BluePippin (Sage Science, Beverly, MA, USA) automated gel extraction. Libraries were validated for concentration and size distribution with Agilent 2100 Bioanalyzer (Agilent, Santa Clara, CA, USA) using a DNA 1000 chip. The resulting libraries were sequenced with Illumina HiSeq 2000 (Illumina, San Diego, CA, USA), each on separate lanes with 100-bp single-end reads.

#### 4.3.2. Variant Detection

PCR and optical duplicate reads were removed prior to sequence processing with BBMap using the *clumpify* function [50]. The duplicated reads had the same sequence at both 5′ and 3′ ends, which were produced by either duplication from the PCR amplification step during the library preparation or during the sequencing process. The reads of similar sequence were sorted together in the process, allowing better compression of the remaining reads. This also allows faster sequence processing and more accurate variant calling. Sequence demultiplexing was done using GBSX software [51] identifying barcode indices with 0 mismatches followed by *KpnI* cut sites, producing individual FastQ files for each sample and clipping out the barcode sequences. FastQ files of each parental line from three lanes were concatenated together. Trimmomatic [52] was used to filter reads shorter than 36 bp, trim the start or end of reads having quality lower than 5 (>0.31 probability error), and clipping Illumina adapter sequences by allowing 2 mismatches in the initial 16 bp seed sequence and entirely clipping the adapter when a matching score reaches 10 (about 17 bp match). The resulting sequences were aligned to rice reference genome [53], *Oryza sativa* L. ssp. *japonica* cv. Nipponbare, using the sequence aligner, Burrows-Wheeler Alignment maximal exact matches [54]. The aligned reads were sorted and assigned groups using Picard [55]. Genome Analysis Toolkit (GATK) *BaseRecalibrator* [56] was performed on the alignment files using initially called homozygous variant sites between the two parental lines as known sites. Genomic variants for each sample were identified using GATK *HaplotypeCaller* and stored into an instance of GenomicsDB datastore [57]. Variants for all samples were extracted from the datastore into VCF files using GATK *GenotypeVCFs* command.

#### 4.3.3. Variant and Sample Filtering

Variant data from restriction enzyme-based genotyping was filtered before adapting genotype data to F_2_ population genetic analysis by following some steps from Furuta et al. [41]. To simplify further analysis, only SNPs with exactly 2 allele counts were selected using BCFtools [58]. Genotype calls with less than 10 allele depth (read count) or less than 30-phred genotyping quality were set to missing calls using VCFtools [59].

SNPs were further filtered by selecting homozygous and polymorphic SNPs between the two parents using Trait Analysis by aSSociation, Evolution and Linkage (TASSEL) [60]. SNPs that have less than 0.18 minimum allele frequencies, and heterozygous proportions less than 0.05 and greater than 0.55 were excluded from the dataset. Redundant SNPs that were found on the same read, within 96 bp from one another, were removed using the *thin* parameter of VCFtools. Samples with at least call rate of 50% and markers with call rate of at least 60% were selected using TASSEL and were used for imputation and genotyping call correction.

#### 4.3.4. Adapting genotyping Data for F_2_ Genetics Analysis

Prior to QTL mapping, adapting to genotyping data for sequencing-based genotyping was done as described by Furuta et al. [41]. “ABH Genotype” file was generated using TASSEL by setting IR64 as parent A and ASD1 as parent B. The genotype file was loaded into RStudio [61] and adjusted using the *ABHgenotypeR* R package [47,62]. Missing data was imputed then corrected for heterozygous and homozygous calls by 10 and 7 bp lengths, respectively. Two additional steps were done further to reduce potentially erroneous calls that were left out by the previous steps. The first was to set remaining double recombinants to missing (e.g., in the sequence HH**AA**BB, AA were replaced by missing data, NN), and the second was to set erroneous calls at the start and end of chromosomes to missing (e.g., in the chromosome start sequence of 5′-**AA**HH or end sequence BB**AA**-3′, both AAs were replaced by NN). These two steps were done by correcting all observed patterns with ≤ 4 bp in length using the *gtmask* R package that we have written (https://github.com/johncarlosignacio/gtmask, accessed on 3 April 2021).

### 4.4. QTL Analysis

#### 4.4.1. Genetic Map Estimation

The output from *ABHgenotypeR* was loaded as cross data of *R/qtl* package [63]. Duplicate markers with the same non-missing genotype calls and adjacent were removed so that only one marker with the same genotype remains. This was done by using the *R/qtl findDupMarkers* function with the parameters *exact.only* set to false and *adjacent.only* set to true. The genetic map was estimated using the *R*/*qtl est.map* function with Kosambi as mapping function.

#### 4.4.2. QTL Detection Using QGene Software

The final mapping dataset was obtained by intersecting the F_2:3_ families that had both phenotypic and genotypic data. The intersection between these two datasets was used for QTL analysis. Interval mapping (IM) and composite interval mapping (CIM) analyses were first done using QGene [42]. QTL scan interval was set to 1.0 cM for both analyses. For CIM, cofactors were selected using stepwise cofactor selection as the method with maximum number of cofactors set to auto, F to add set to 0.01, and F to drop set to 0.01. Thresholds for 0.05 and 0.01 significances were obtained using 10,000 permutations.

#### 4.4.3. QTL Setection Using QTL Cartographer Software

The data was also analyzed using WinQTL Cartographer for comparison [43]. For both IM and CIM analyses, walk speed was set to 1.0 cM. For CIM, the model used was no. 6 (standard model), automatically selecting 5 control markers, setting window size to 1.0 cM, and forward & backward regression probabilities for into and out of 0.01. Thresholds for 0.05 and 0.01 significances were obtained using 1000 permutations.

### 4.5. QTL Comparisons

Detected QTLs in this study were compared to previously published QTLs to determine whether the detected QTLs were novel. If previous studies presented genetic map information, the map positions were converted into physical positions by querying the corresponding primer sequences against the rice reference genome [44] with nucleotide basic local alignment search tool (BLAST) [64].

## Figures and Tables

**Table 1:** Summary of constructed genetic map of F_2:3_ mapping population based on 1921 markers and 200 F_2:3_ families.

Chr.	No. of Markers	Length (cM)	Ave. Interval (cM)	Max Interval (cM)
1	202	199.9	1	9.9
2	193	189.6	1	5.7
3	191	179.4	0.9	11.8
4	128	132.3	1	9.6
5	136	141	1	6.5
6	166	156.3	0.9	9.8
7	161	154.6	1	5.2
8	212	160.2	0.8	6.9
9	116	107.6	0.9	6.2
10	134	97.5	0.7	6.6
11	127	145.1	1.2	21.2
12	155	128.3	0.8	7.4
overall	1921	1791.5	0.9	21.2

**Table 2:** QTLs for anaerobic germination identified in IR64/ASD1 mapping population.

QTL	Chr.	Markers			QGene IM			QGene CIM			QTL Cart IM			QTL Cart CIM		
		Left flank	Peak	Right flank	LOD	*R^2^*	Add	LOD	*R^2^*	Add	LOD	*R^2^*	Add	LOD	*R^2^*	Add
*qAG5*	5	S05_7886279	S05_9596301	S05_14907902	-	-	-	*2.1 ^a^*	*5.4*	*2.2*	*2.1*	*0.0*	*0.0*	*2.9*	*0.0*	*−0.5*
*qAG6*	6	S06_10782720	S06_11109090	S06_11200565	*2.3*	*5.1*	*5.1*	*2.2*	*5.0*	*2.4*	*2.6*	*2.7*	*3.9*	*2.6*	*0.3*	*1.3*
*qAG7*	7	S07_6302957	S07_7547815	S07_7650364	5.6	12.1	8.4	7.1	15.1	8.0	5.5	11.7	8.0	7.5	10.7	8.0
*qAG9*	9	S09_12029625	S09_12277535	S09_12331208	12.0	24.3	13.0	15.0	29.4	13.0	12.0	30.2	14.0	14.5	27.6	15.0

^a^ Italicized logarithm of the odds (LOD) scores were below the significant permutation threshold, but with LOD >2

**Table 3:** Previously reported QTL for anaerobic germination that overlaps with *qAG9*.

QTL	Source	Marker Position (Mb)			Range (Mb)	Marker	Software	IM		CIM	
		Left Flank	Peak	Right Flank				LOD	R^2^	LOD	R^2^
*qAG-9-2*	Khao Hlan On	11.75	12.31	12.55	0.80	136 SSR	QTLCart	20.3	33.5	15.3	20.6
*qAG9* ^a^	ASD1	12.03	12.28	12.33	0.30	RAD-seq	QGene	12.0	24.3	15.0	29.4

^a^ This QTL was identified in the current study.

**Table 4:** Previously reported QTLs for anaerobic germination that overlap with *qAG7*.

QTL	Source	Marker Position (Mb)			Range(Mb)	Marker	Software	IM		CIM	
		Left Flank	Peak	Right Flank				LOD	R^2^	LOD	R^2^
*qAG7.1*	Ma-Zhan Red	8.04	9.07	12.97	4.93	118 SSR, indel	QGene	13.7	30.3	14.5	31.7
*qAGS-7*	TKM 9	7.61	11.09	12.78	5.17	91 SSR	QGene	7.8	11.1	7.6	17.8
*qAG-7-1*	Khao Hlan On	6.07	10.37	17.67	11.60	136 SSR	QTLCart	5.1	9.9	-	-
*qAG7.1*	Kharsu 80A	3.68	4.80	6.01	2.32	BeadXpress	QGene	5.5	17.5	4.8	15.4
*qAG7*	Nanhi	4.80	NA^a^	18.48	13.68	BeadXpress	QTLCart	11.2	11.7	13.9	14.1
*qSUR7–1_Rc238-SCR-21_*	Kalarata	NA	6.07	NA	NA	KASP	IciM	20.3	39.7	NA	NA
*qAG7* ^b^	ASD1	6.30	7.55	7.65	1.35	RAD-seq	QGene	5.6	12.1	7.1	15.1

^a^ Not applicable (no data available); ^b^ This QTL was identified in the current study.

## Data Availability

The genotype data of the mapping population is accessible in the *gtmask* R package (https://github.com/johncarlosignacio/gtmask, accessed on 3 April 2021).

## Supplementary Materials

The following are available online at https://www.mdpi.com/article/10.3390/plants10040705/s1, Figure S1: Representative tray showing different levels of survival rates, Figure S2: Plot of LOD from interval mapping QTL analysis of F_2:3_ mapping population derived from IR64 and ASD1 on chromosomes 5, 6, 7, and 9 from WinQTL Cartographer, Figure S3: Plot of LOD from composite interval mapping QTL analysis of F_2:3_ mapping population derived from IR64 and ASD1 on chromosomes 5, 6, 7, and 9 from WinQTL Cartographer, Table S1: Distribution of genotypes at each data correction step.
