# Supplementary material for: Genetic Mapping by Sequencing More Precisely Detects Loci Responsible for Anaerobic Germination Tolerance in Rice
Source: Plants (Basel). 2021 Apr 6;10(4):705. doi: 10.3390/plants10040705 (PMC8067528; doi:10.3390/plants10040705)
Supplement: Supplementary file 1 [file plants-10-00705-s001.pdf]

Supplementary Materials

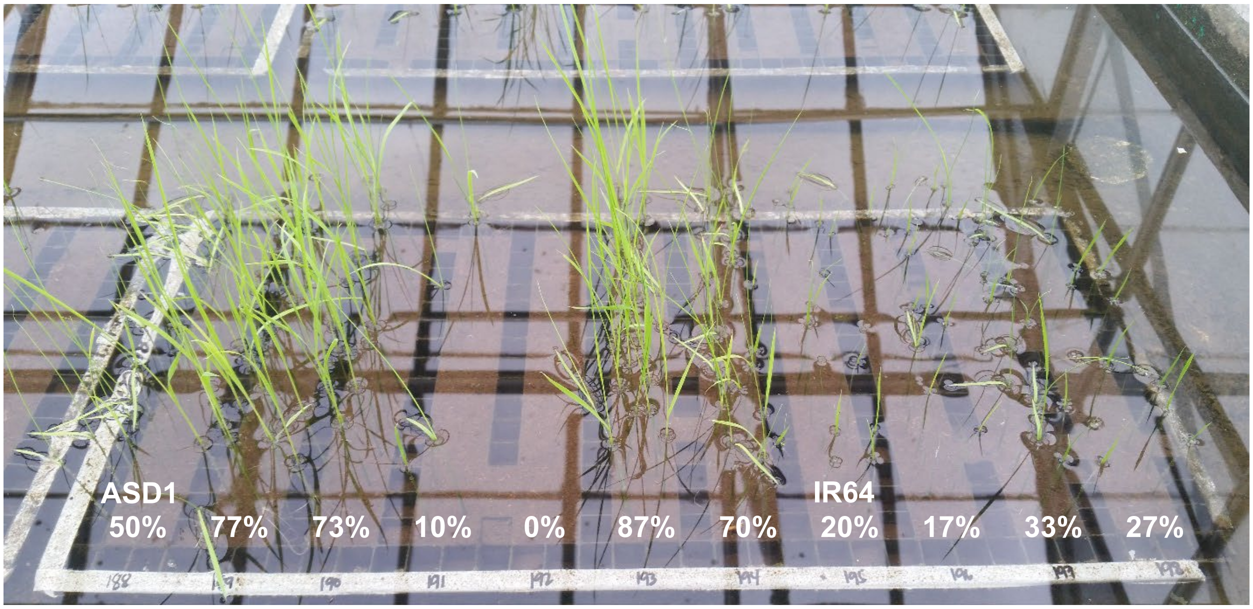

Figure S1. Representative tray showing different levels of survival rates of IR64 (20%), ASD1 (50%), and 9 of their $F_{2:3}$ progenies (0% to 87%) under anaerobic germination stress at 14 days after seeding.

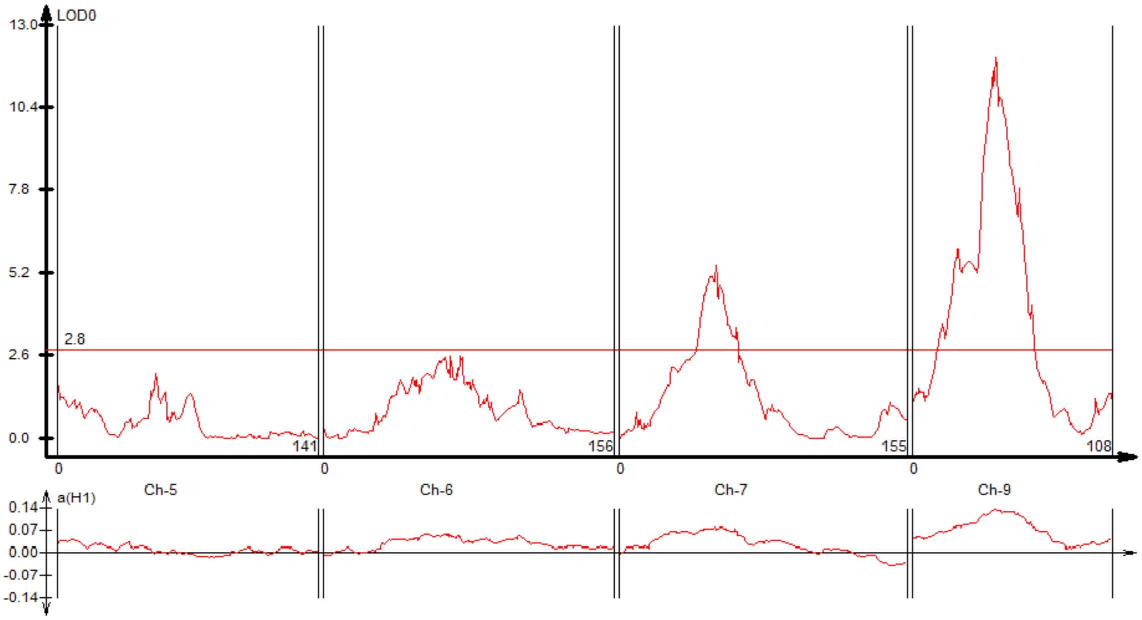

Figure S2. Plot of LOD from interval mapping QTL analysis of $F_{2:3}$ mapping population derived from IR64 and ASD1 on chromosomes 5, 6, 7 and 9 from WinQTL Cartographer

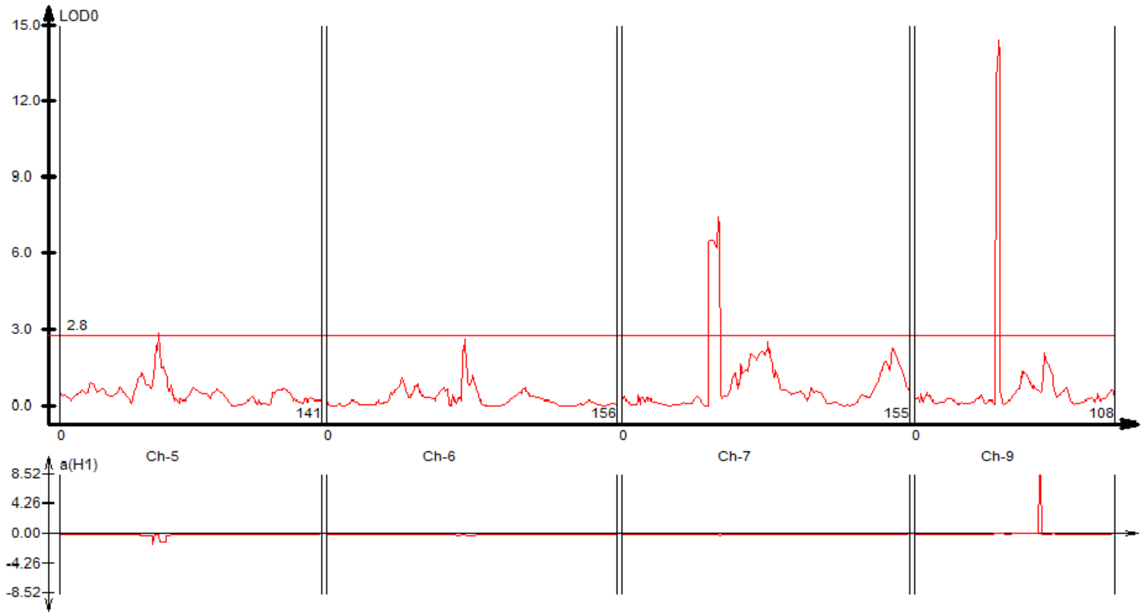

Figure S3. Plot of LOD from composite interval mapping QTL analysis of $F_{2:3}$ mapping population derived from IR64 and ASD1 on chromosomes 5, 6, 7 and 9 from WinQTL Cartographer

Table S1. Distribution of genotypes at each data correction step

Step	Length corrected	% A	% B	% H	% N
Input	-	27.41	24.80	27.42	20.38
Imputation of missing data	Indefinite	32.46	29.40	32.02	6.12
Correct under-called heterozygous	≤ 10 bp	26.71	23.59	48.30	1.40
Correct homozygous stretches	≤ 7 bp	27.42	23.62	47.96	1.00
Mask double recombination	≤ 4 bp	27.35	23.51	47.96	1.19
Mask chromosome ends	≤ 4 bp	27.27	23.43	47.92	1.38
